# The King's Death

**Published:** 1910-05-21

**Authors:** 


### THE KING'S DEATH.

### The Lying-in-State.

### THE NATION'S LAST TRIBUTE.

As we go to press King Edward VII. is lying in state in Westminster Hall, and many hundreds of thousands of his subjects are paying silently their last homage to the dead Sovereign by filing past his bier. There in the old hall stands the purple-draped catafalque flanked by the laurel wreaths and guarded by Yeomen, Gentlemen, Grenadiers, and Ghoorkhas. At its foot is a pile of flowers, the scent of which faintly perfumes the hall. All day long on Wednesday and Thursday, and all the afternoon on Tuesday, the public trooped in double file past the pall-covered coffin bearing the Royal insignia. On Tuesday morning the procession from Buckingham Palace to the hall had attracted vast crowds that lined the roadway all along the route. That was, perhaps, the attraction of a grand spectacular effect; this, the silent passage through the felt-carpeted hall, is a tribute of respect in which morbid curiosity and the mere desire of sight-seeing have no part, for there is little to see that is morbid, and the stage effect is largely diminished by the twilight of the place and the sombreness of the surroundings. No more striking evidence of the love and respect which the people bore for Edward VII. can be imagined than that vast queue, stretching, miles in length, from Dean's Yard to Vauxhall.

An address of condolence has been sent on behalf of the League of Mercy to his Majesty the King, George V., by the Executive Council.

In consequence of the death of King Edward, the public presentation of graduates of the London University has been postponed until Foundation Day, Monday, November 28. Invitations will be issued in due course.

We have received from Messrs. Eyre and Spottiswoode, of 33 Paternoster Row, a copy of the new Prayer-book, in which the alterations authorised by the Order in Council, dated May 10, and also the Royal Warrant from the Home Office authorising the new Accession Service, have been duly embodied. The editions hitherto published by the Oxford and Cambridge Presses have not included the latter authority.

The Duke of Northumberland presided at an extraordinary meeting of the Court of Governors of Middlesex Hospital, when the following addresses to King George and Queen Alexandra were passed :

To the King's most excellent Majesty : the humble address of the president, vice-presidents, treasurers, weekly board, medical staff, and governors of the Middlesex Hospital : "Most gracious Sovereign,—We, your Majesty's dutiful and loyal subjects, humbly venture to approach your Royal presence to offer our respectful sympathy and to express our heartfelt sorrow at the lamented death of your Majesty's august father, our late Sovereign Lord, King Edward. We fully share the profound grief into which the whole Empire is plunged by the irreparable loss which your Majesty has sustained, and we humbly trust that the universal tribute of respect and reverence paid to the memory of our beloved Sovereign may comfort and console your Majesty in your bereavement. We wish to record our deep sense of gratitude for the special mark of his Royal favour in graciously consenting to act as the patron of this hospital throughout the entire period of his wise and beneficent reign. We particularly desire to express our high appreciation of the many munificent gifts which his late Majesty bestowed upon this hospital, whilst we take especial pride in the reflection that it was largely due to the sympathy and interest which his Majesty displayed in every effort made for the alleviation of suffering and the encouragement of scientific research that the Middlesex Hospital Cancer Charity was established upon its present basis. We recall with feelings of fervent admiration the noble work which his Majesty carried out by initiating King Edward's Hospital Fund for London, and later, by his continued influence, consolidating it into an organisation which has already more than fulfilled the purpose it was designed to serve. We further humbly crave leave respectfully to congratulate your Majesty on your accession to the throne of your ancestors. We assure your Majesty of our unalterable attachment and devotion to your person, and we fervently pray that your Majesty may long be spared to guide the destinies of this realm."

To her Majesty Queen Alexandra : the humble address of the president, vice-presidents, treasurers, weekly board, medical staff, and governors of the Middlesex Hospital : "Your gracious Majesty,—We humbly and respectfully venture to tender to your Majesty an expression of our profound and abiding sorrow at the death of his Majesty King Edward VII., of blessed and glorious memory, and to assure your Majesty of the sincere and heartfelt sympathy we feel for your Majesty in your Majesty's grievous affliction and loss."

The following resolution was passed at the weekly Meeting of the Board of Governors of the General Infirmary, Leeds, Mr. Charles Lupton in the Chair : The Board of the General Infirmary at Leeds desire to convey to his Majesty King George the Fifth, her Majesty Queen Mary, and her Majesty Alexandra the Queen Mother, and the members of the Royal family, their deep and heartfelt sympathy at the irreparable loss which they and the nation have sustained by the death of King Edward. The Board recall with gratitude his late Majesty's visit to Leeds to open their Hospital in 1868, as well as the keen interest which he displayed in Hospitals throughout the country. In conclusion, they express the hope that their Majesties King George and Queen Mary may be blessed with a long and prosperous reign.

At a special meeting of the President and Fellows of the Royal College of Physicians of Ireland, held on Wednesday, May 11, the following resolution was proposed by Sir Francis Cruise, seconded by Dr. Little, and carried unanimously, all present standing : "Resolved—The President and Fellows of the Royal College of Physicians of Ireland, in College Hall assembled, whilst expressing their condolence and deepest sympathy with his Majesty George V. upon the death of his late Majesty the beloved King Edward VII., his father, desire to assure his Majesty of their loyal devotion to his Throne and person, of their earnest desire that his reign may be as illustrious and beneficent as that of the great Sovereign whom he succeeds, and of their fervent hope that his life may long be spared to enable him to work for the good and amelioration of his people." Before the proceedings terminated the assembled Fellows took the Oath of Allegiance to his Majesty King George V.

In view of the lamented death of his Majesty King Edward VII., the Conference of the Child Study Society, arranged to be held at Tunbridge Wells on May 19, 20, and 21, has been postponed to June 10 and 11. Copies of rearranged programme can be had on application to Miss Watt, 35 Church Road, Tunbridge Wells.

At the meeting of the board of the Royal Free Hospital, of which his late Majesty was patron, Major Ricardo, the chairman, referred to the great loss which had been sustained by the death of his Majesty King Edward VII., and moved the following address to his Majesty King George V.: "To the King's most Excellent Majesty,—We, your Majesty's most loyal and dutiful subjects, respectfully beg leave to express our heartfelt sorrow at the loss sustained by your Majesty, the Royal Family, and the Empire by the death of your Majesty's revered father, our late most gracious and beloved Sovereign and patron of this institution, and also to express our dutiful congratulations upon your Majesty's accession to the Throne, as well as our sincere attachment to your Royal person." The following resolution was passed for forwarding to the Queen Mother: "To her Majesty Queen Alexandra,—That the board of the Royal Free Hospital record their profound sorrow at the death of his most gracious Majesty King Edward VII., who as Prince of Wales became the vice-patron of the hospital in 1863, and in 1895 accompanied your Majesty when publicly opening the new 'Alexandra' wing of this hospital. Upon his accession to the throne his late Majesty graciously confirmed his Royal favour and became the patron of the hospital. The board respectfully tender to your Majesty their heartfelt sorrow on the irreparable loss which your Majesty and the Royal Family have sustained."

The Council of the Royal College of Surgeons on May 12 passed the two following addresses of condolence, respectively, to his Majesty the King and to her Majesty Queen Alexandra: "May it please your Majesty,—We, the President, Vice-Presidents, and Council of the Royal College of Surgeons of England, venture to approach your Majesty in order to express our deep grief at the sudden and unexpected death of your Majesty's beloved father, our late revered Sovereign, and we humbly pray your Majesty graciously to receive our respectful sympathy. During the reign, which was all too short, he has gained the love and confidence of his subjects throughout the world to a degree which we believe has never been surpassed by any Sovereign. At the same time he endeared himself to the people of foreign countries in a remarkable manner by his tact, courtesy, and kindness, so that his death will be felt by them in little less degree than by his own subjects. Surely no monarch was ever more sincerely mourned than he will be. We desire respectfully to express our devoted loyalty to your Majesty, and our trust that God may grant you good health, and that your Majesty may live to reign for many years in the hearts of your people." "May it please your Majesty,—We, the President, Vice-Presidents, and Council desire humbly to offer our sincere and respectful condolence with your Majesty under the great loss which your Majesty and your illustrious family have sustained by the sudden and unexpected death of his Majesty King Edward VII. We earnestly and respectfully trust that the expressions of grief which have been sent from every part of the world will help to mitigate, in some degree, your Majesty's private sorrow."

The staffs of the various hospitals with which the late King was connected as patron have sent wreaths to Windsor.

Mr. Thomas Bryant, as one of the late King's honorary surgeons, was unavoidably prevented from taking part in the funeral procession from Buckingham Palace to Westminster Hall last Tuesday.

Nine matrons of the leading London hospitals have united with their nursing staffs and sent a wreath to the bier of the late King. The wreath is diamond-shaped, composed of palms, laurestinas, orchids, purple irises, and white heather, and with a royal-purple ribbon bearing the following inscription in silver letters: "With profound sorrow, from the matrons and nursing staffs of St. Thomas's, St. George's, Middlesex, St. Mary's, University College, King's College, Royal Free, Charing Cross, and Westminster Hospitals."

At the meeting of Convocation of the University of London on Tuesday last Sir Edward Busk, on taking the chair, moved that an address of condolence and congratulation to King George be presented by Convocation, and that they concur with the Senate's resolution of sorrow and respectful sympathy with the Queen Mother. King Edward's connection with the University has been vital. He was the Visitor of the University, and it was his duty to examine into the way in which it had done the work which the Legislature entrusted to it. He has been Visitor during the whole of his reign, when the creation of the teaching side of the University and its development has taken place. The resolution was carried unanimously, all those present rising.

At a special meeting of the Executive Committee of King Edward's Hospital Fund for London the following resolutions were unanimously adopted: That the Executive Committee of King Edward's Hospital Fund for London, on behalf of the General Council, desire to record their profound sorrow at the great loss which all those associated with the Fund have sustained by the death of their beloved Sovereign, King Edward VII. It was on his initiative that the commemoration of the sixtieth year of the reign of Queen Victoria took the form of a scheme for the assistance of the hospitals of London, and the Fund which he thus founded and which bears his name is, therefore, a lasting monument to his constant sympathy with the poor and suffering. To his active personal share in its administration during the years in which he was president the Fund owed its rapid establishment in public confidence, and he gave repeated assurances of the interest with which he watched its continued progress under his illustrious successor. The Committee respectfully desire to tender their deepest sympathy to his Majesty King George V. and his gracious Consort in the sad bereavement sustained by their Majesties, and to offer the humble assurance of their loyalty and devotion. That the Executive Committee of King Edward's Hospital Fund for London desire in the name of the General Council respectfully to tender to her Majesty the Queen-Mother their deepest sympathy in the immeasurable loss which she has sustained in the death of his Majesty King Edward VII. The Committee feel sure that her Majesty, who has herself taken so great an interest in hospitals, will appreciate the great loss which the Fund has sustained in the death of its founder and its first president.

